# Mussel-Inspired Redox-Active and Hydrophilic Conductive Polymer Nanoparticles for Adhesive Hydrogel Bioelectronics

**DOI:** 10.1007/s40820-020-00507-0

**Published:** 2020-08-18

**Authors:** Donglin Gan, Tao Shuai, Xiao Wang, Ziqiang Huang, Fuzeng Ren, Liming Fang, Kefeng Wang, Chaoming Xie, Xiong Lu

**Affiliations:** 1 Key Lab of Advanced Technologies of Materials, Ministry of Education, School of Materials Science and Engineering, Southwest Jiaotong University, Chengdu, 610031 People’s Republic of China; 2 Department of Materials Science and Engineering, Southern University of Science and Technology, Shenzhen, 518055 Guangdong People’s Republic of China; 3 Department of Polymer Science and Engineering, School of Materials Science and Engineering, South China University of Technology, Guangzhou, People’s Republic of China; 4 National Engineering Research Center for Biomaterials, Sichuan University, Chengdu, 610064 People’s Republic of China

**Keywords:** Mussel-inspired, Redox-active nanoparticles, Conductive polymer, Conductive hydrogel, Adhesive bioelectronics

## Abstract

Conductive polymers (CPs) are generally insoluble, and developing hydrophilic CPs is significant to broaden the applications of CPs. In this work, a mussel-inspired strategy was proposed to construct hydrophilic CP nanoparticles (CP NPs), while endowing the CP NPs with redox activity and biocompatibility. This is a universal strategy applicable for a series of CPs, including polyaniline, polypyrrole, and poly(3,4-ethylenedioxythiophene). The catechol/quinone contained sulfonated lignin (LS) was doped into various CPs to form CP/LS NPs with hydrophilicity, conductivity, and redox activity. These CP/LS NPs were used as versatile nanofillers to prepare the conductive hydrogels with long-term adhesiveness. The CP/LS NPs-incorporated hydrogels have a good conductivity because of the uniform distribution of the hydrophilic NPs in the hydrogel network, forming a well-connected electric path. The hydrogel exhibits long-term adhesiveness, which is attributed to the mussel-inspired dynamic redox balance of catechol/quinone groups on the CP/LS NPs. This conductive and adhesive hydrogel shows good electroactivity and biocompatibility and therefore has broad applications in electrostimulation of tissue regeneration and implantable bioelectronics.

**Electronic supplementary material:**

The online version of this article (10.1007/s40820-020-00507-0) contains supplementary material, which is available to authorized users.

## Highlights

- A universal strategy was proposed to producing conductive, redox-active, and hydrophilic sulfonated lignin-conductive polymer nanoparticles (CP/LS NPs).
- By incorporating the CP/LS NPs into hydrogel network, a good conductive, adhesive, and tough hydrogel was obtained.
- The redox-active NPs maintained enough catechol groups inner the hydrogel for adhesiveness.

**Electronic supplementary material:**

The online version of this article (10.1007/s40820-020-00507-0) contains supplementary material, which is available to authorized users.

## Graphical abstract

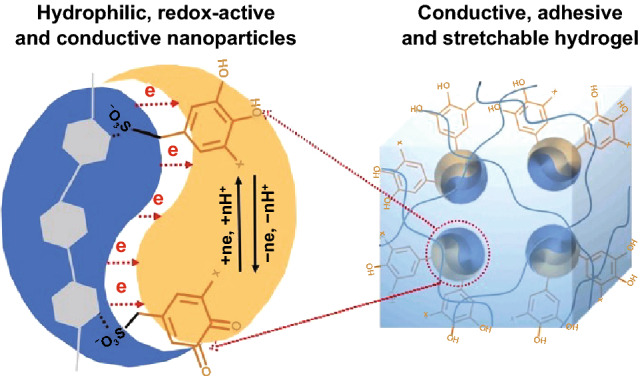

## Introduction

Conductive hydrogels (CHs) are an emerging class of hydrogels that combine biocompatibility and conductivity. These properties make CHs useful in bioelectronics. CHs are generally prepared by filling a hydrogel matrix with conductive materials such as graphene [1–4], carbon nanotubes [5, 6], metallic nanoparticles (NPs) [7], and organic or inorganic salts [8]. For example, Zhang et al. [9] prepared cellulose nanofibers and graphene co-incorporated poly (vinyl alcohol)-borax (GN-CNF@PVA) hydrogel, which had good mechanical flexibility, strength, and conductivity. Han et al. [5] used PDA-chelated CNT-Fe_3_O_4_ nanohybrids to construct an anisotropic hydrogel, which possessed conductive, magnetic, and self-adhesive properties. In particular, CPs, such as polyaniline (PANI), polypyrrole (PPY), and poly(3,4-ethylenedioxythiophene) (PEDOT), are promising conductive fillers for CHs because they have good conductivity and flexibility [10–13]. However, there are scientific challenges for using CPs as fillers to preparing CHs in biomedical applications. First, CPs generally have poor water solubility, and hydrophobic CPs cannot be well dispersed and integrated with the hydrophilic hydrogel network. Thus, the mechanical properties and conductivity of reported CP-based CHs are generally weak. The typical approach to produce water-soluble CPs is to complex them with other hydrophilic molecules. For example, PEDOT is often doped with hydrophilic poly(styrene sulfonate) (PSS) to improve its conductivity [14, 15]. Unfortunately, the high content of PSS results in an acidic physiological environment, which restricts the long-term use of PSS-doped PEDOT in clinical practice [16]. Second, the biocompatibility of many CPs is insufficient and reported CP-based CHs lack cell affinity and biocompatibility [17]. Third, most modern bioelectronics are designed to be conformable and to tightly integrate with surrounding tissue [18, 19]. However, CP-based CHs generally lack tissue adhesiveness and the interfacial adhesion between CHs and tissue is weak, resulting in high interfacial resistance and unstable electrical signals. Thus, a novel strategy for fabricating hydrophilic and biocompatible CP fillers is therefore required for developing adhesive and conductive hydrogels applying in bioelectronics.

Adhesive hydrogels can be realized by tuning the chemical bonding and mechanics of energy dissipation [20]. Various adhesive hydrogels have been reported using different adhesion strategies, such as host–guest [21–23], nucleobase [24], and energy dissipative matrix [25]. Adhesive hydrogels could also come from biomolecules or biopolymers. Gao et al. [26] reported an adhesive polyacrylamide hydrogel driven by lysine, which exhibited excellent adhesiveness on different substrates. Wei et al. [27] designed chitosan–silicotungstic acid–polyacrylamide with repeatable adhesive capacity and highly sensitive conductivity upon strain, which demonstrated great potential for wearable strain sensors. In particular, adhesive hydrogels could be designed by learning from natural adhesion mechanisms such as those found in sundew [28] and sandcastle worms [29]. Recently, adhesive hydrogels based on mussel-inspired catechol chemistry have attracted much attention [30, 31]. The adhesiveness of catechol-based hydrogels is attributed to the covalent/noncovalent reactions between the catechol groups of the hydrogel and substrate [32–34]. Mussels retain their long-term adhesion properties because of the redox balance between quinone and catechol groups, which is achieved by secreting reductive and oxidative proteins in their byssal thread [35, 36]. We previously demonstrated that manipulating the redox balance of quinone/catechol groups of polydopamine (PDA) could endow hydrogels with long-term adhesiveness [2, 37, 38]. The adhesion mechanism of mussels inspired us to develop catechol chemistry-based hydrogels with high conductivity and long-term adhesiveness.

Lignin and its derivatives are abundant, renewable, and environmentally friendly natural polymers isolated from plants. Lignin has complex and varying structures with numerous functionality such as hydroxyl, methoxy, and phenolic hydroxyl groups [39, 40]. Lignin can be sulfonated to obtain water-soluble sulfonated lignin, which has a high content of sulfonate groups [41]. These negatively charged sulfonate groups can act as charge balancing counterions to positively charged CP chains. Thus, water-soluble sulfonated lignin can be used to complex with polymers, which not only improve the hydrophilicity, but also act as a dopant to improve the conductivity of the polymer. LS has been used as a dispersant or dopant to improve the dispersibility and conductivity of graphene [42], PANI [43], and PEDOT [44]. In particular, LS exhibits redox activity because it contains abundant oxidative quinone groups [45]. We previously reported that lignin complexation with Ag formed a dynamic redox environment based on quinone/catechol couples, and this endowed the hydrogel with long-term and repeatable adhesiveness [46].

**Fig. 1:**
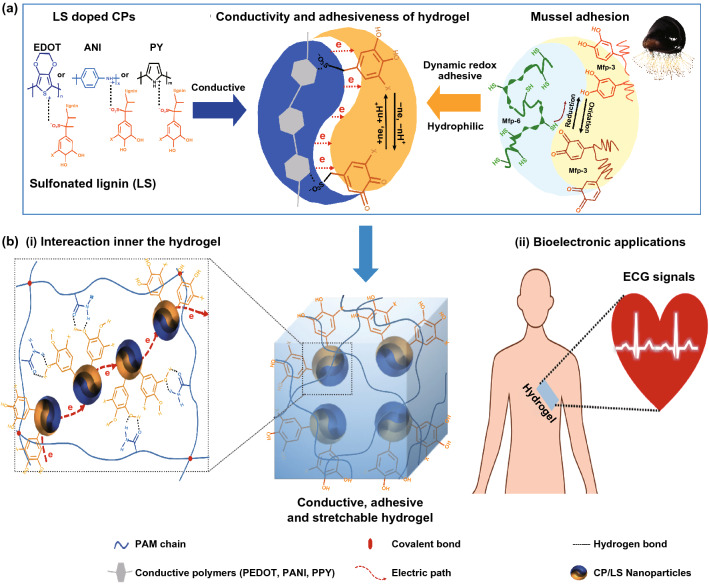
Preparation of hydrophilic and redox-active conductive polymer/sulfonated lignin (CP/LS) NPs-incorporated conductive and adhesive hydrogels. **a** Conductive and adhesive mechanisms of the hydrogel. Left: LS-doped CPs, such as PEDOT, PANI, and PPY. Right: mussel adhesion mechanism. **b** CP/LS NPs were incorporated into the hydrogel network to obtain a hydrogel with high conductivity and adhesiveness. (i) The NPs can form physical interaction with the Polyacrylamide (PAM) network and construct conductive pathway inner the hydrogel. (ii) The CH is adhesive and therefore is compatible with human tissue for bioelectronic applications

In this study, a mussel-inspired strategy was designed to construct redox-active, hydrophilic conductive NPs by using LS as dopant to CP (Fig. 1a left). One part of LS contains abundant sulfonate groups that are doped into the CP to promote conductivity. Another part of LS provides catechol groups to improve the hydrophilicity and redox activity of the CP (Fig. 1a right). Subsequently, the CP/LS NPs were used as versatile nanofillers to incorporate in hydrogels and endow the hydrogels with good conductivity and adhesiveness. The CP/LS NPs-based conductive and adhesive hydrogels were potent to be used for bioelectronic applications (Fig. 1b).

## Experimental Section

### Materials

Alkali lignin (Wn = 1000−10,000) was purchased from Qunlin paper Group Co., China. 3,4-Ethylenedioxythiophene (EDOT), pyrrole (PY), aniline (ANI) were supplied by Macklin. Ammonium persulfate (APS), sodium hydroxide (NaOH), N,N’-methylenebisacrylamide (BIS), N,N,N’,N’-tetramethylethylenediamine (TMEDA), and acrylamide (AM) were purchased from KESHI Chemical Works in Chengdu.

### Preparation of CP/LS NPs

The CP/LS NPs were prepared using the following procedure. Firstly, lignin was sulfonated by APS according to the previous report to prepare LS solution [41, 42]. An CP/ethanol solution with different concentrations was added into the LS solution (0.15 wt%) under vigorous stirring for 20 min until the uniform dispersion of CP. Then, an APS (1.5 times the weight of the CP) solution was added dropwise to the LS-CP solution. The resulting mixture was stirred for 48 h in an ice bath (4 °C) to polymerize CP and complexed with LS. CP/LS was obtained by centrifuging and washing the resulting mixture with water and ethanol several times. Three kinds of conductive polymer (CP), such as PEDOT, PPY, and PANI, were used for preparation of CP/LS NPs. The compositions of the CP/LS are listed in Table S1.

### Preparation of CP/LS-PAM Hydrogels

The hydrogels were synthesized using the following procedure. AM, ammonium persulfate (APS), N, N-methylenebisacrylamide, and TMEDA were added in a breaker placed in an ice bath. The CP/LS-PAM hydrogel was synthesized after stirring the reaction mixture for 10 min. Hydrogels with different CP to LS and CP/LS to AM mass ratios were prepared. The compositions of the hydrogels are listed in Table S2.

### Characterization of the Hydrogels

The freeze-dried PEDOT-PAM and PEDOT/LS-PAM hydrogels were then broken apart, and their inner morphologies were observed by SEM (JSM 6300, JEOL, Japan). The mechanical properties and adhesive strength of the PAM, PEDOT-PAM, and PEDOT/LS-PAM hydrogels were measured by carrying out their tensile adhesive tests using a universal testing machine (Instron 5567, USA), according to a previously reported procedure [2]. The conductivity of the hydrogel was measured by two-probe method on an electrochemical system (CHI 660, Chenghua, China). The biocompatibility of the hydrogel is evaluated in vitro and in vivo. All the animal experiments were performed according to the protocols approved by the local ethical committee and the laboratory animal administration rules of China. Details of the characterizations are described in the supplemental information.

## Results and Discussion

### Design Strategy

To overcome the hydrophobic property of CPs, the conductive, redox-active, and hydrophilic CP/LS NPs were prepared by a universal method. The NPs had good conductivity because the negatively charged LS acted as counter ions to be doped into the CP and increase its conductivity. The NPs had excellent water dispersibility because of the hydrophilic catechol and sulfonate groups of LS. The NPs had redox activity because of the catechol/quinone groups on the lignin molecules. During the lignin sulfonation process, the addition of ammonium persulfate (APS) facilitated the grafting of sulfonated groups to lignin and oxidized the catechol groups to quinone groups (Fig. S1). During the process of the CP polymerization, the CP changed from a reduced state (intrinsic state) to a partially oxidized state, while electrons were transferred from the CP to LS, thereby converting the quinone groups into catechol groups (Fig. 1a, b). In short, the LS and CP formed an electron donor–acceptor complex. This complex facilitated electron transfer between catechol and quinone groups, avoiding the excessive oxidization of catechol groups. Thus, it rendered the NPs with abundant catechol groups.

As a nanofiller with multifunctionality, CP/LS NPs were incorporated into the polyacrylamide (PAM) network to obtain adhesive, conductive, and stretchable hydrogels (Fig. 1b). The CP/LS NP-incorporated hydrogel had good conductivity due to the uniform distribution of LS-doped NPs within the hydrogel network, which formed well-connected conductive pathways (Fig. 1b, i). The hydrogel had good mechanical properties due to the nanoreinforcement of NPs, which introduced the noncovalent interactions within the chemically cross-linked PAM hydrogel network (Fig. 1b–i). The hydrogel was biocompatible because the incorporated NPs had cell/tissue affinitive catechol groups. These advantages make the hydrogel suitable for bioelectronics application (Fig. 1b–ii).

The hydrogel had long-term and repeatable adhesiveness because the redox-active NPs formed a dynamic redox system and maintained sufficient catechol groups within the hydrogel. The adhesive mechanism of the hydrogel is similar to that of mussels (Fig. 1a, right). Mussels maintain their long-term adhesiveness because of the dynamic redox reaction of catechol/quinone groups among the mussel foot proteins (Mfp). To avoid excessive oxidation of catechol groups on the Mfp-3 and Mfp-5, the reductive Mfp-6 is secreted by the mussel to maintain the redox balance [47–49]. We previously demonstrated that redox-active lignin promotes hydrogel adhesion [2, 46]. In this hydrogel, the redox-active LS and CP in the NPs formed an electron donor–acceptor complex, which facilitated the dynamic conversion between catechol and quinone groups. In short, the CP/LS NPs created a dynamic redox environment within the hydrogel network, which mimicked that of catechol/quinone groups in mussels and provided abundant catechol groups for hydrogel adhesion.

### Characterization of CP/LS NPs

**Fig. 2:**
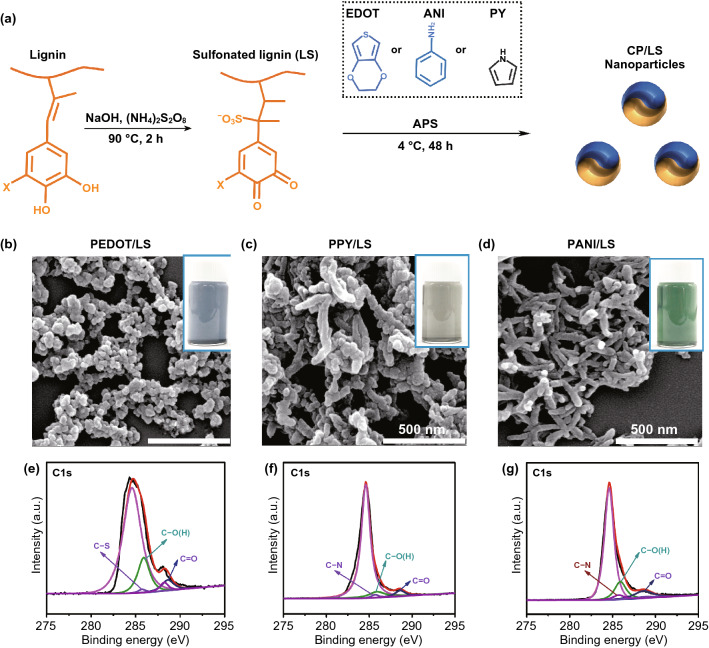
Preparation and characterization of CP/LS NPs. **a** Preparation process of LS and CP/LS NPs. SEM images of **b** PEDOT/LS, **c** PPY/LS, and **d** PANI/LS NPs. The inset images showed the good water dispersibility of the various conductive NPs. XPS analysis of **e** PEDOT/LS, **f** PPY/LS, and **g** PANI/LS NPs

To determine the universality of the LS as a complexation template, three kinds of NPs were prepared, including PEDOT/LS, PPY/LS, and PANI/LS NPs (Fig. 2a). After doped by LS, the three kinds of CP/LS NPs were well dispersed in aqueous solution due to the synergistic contribution of catechol and sulfonate groups of LS. Compared with pristine PEDOT NPs, the LS-doped CP/LS NPs did not agglomerate and/or precipitate after standing as long as 2 days (Figs. S2, S3). These NPs showed different morphologies depending on the type of CP (Fig. 2b–d). PEDOT/LS appeared as spherical NPs, while PPY/LS and PANI/LS had nanorod-like structures. XPS analysis showed that the characteristic peaks of C-S and C-N bonds appeared at 285.15 and 285.53 eV in the PEDOT/LS, PPY/LS, and PANI/LS NPs (Fig. 2e–g). The characteristic XPS peaks of the C–O bonds of the abundant catechol groups appeared at 286.58 eV. These results demonstrated that LS doped into CP, and that the CP/LS NPs contained catechol groups. This was caused by the CP promoting electron transfer between the quinone and catechol groups of lignin, which ensured a dynamic redox balance in the CP/LS NPs. Furthermore, the CP/LS NPs also exhibited the high antioxidative ability to scavenge free radicals (Fig. S4).

### Adhesive Properties of CP/LS NPs-Incorporated Hydrogels

**Fig. 3:**
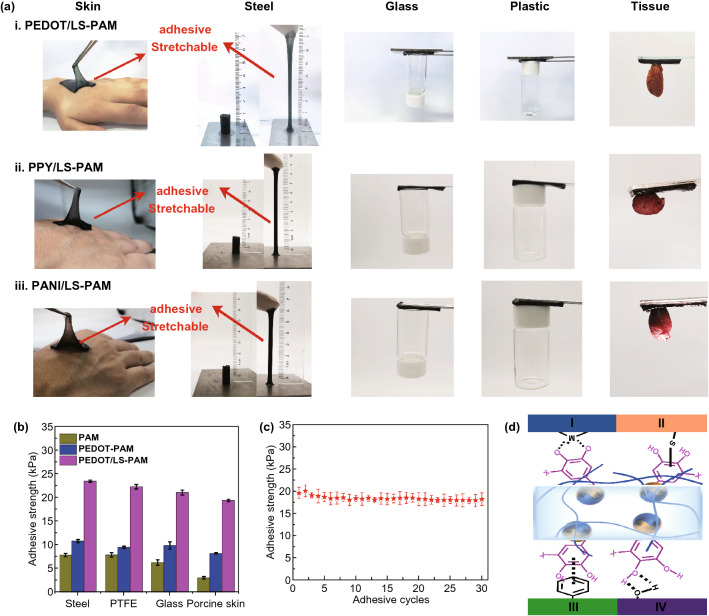
Adhesion of the CP/LS NPs-incorporated hydrogels. **a** CP/LS-PAM hydrogel adhered to skin of hand and steels and was stretched with a large strain; the hydrogel can also adhere to glass, plastic, and fresh tissue from SD rat. **b** Adhesive strength of PEDOT/LS-PAM hydrogel to different substrates. **c** Repeatable and long-term adhesive strength of the PEDOT/LS-PAM hydrogel. (The ratio of EDOT/LS is 2:1, and the content of PEDOT/LS is 3wt ‰.) **d** Interactions between the hydrogel and various substrates; I. coordination bond. II. Covalent linking. III. π–π interaction. IV. Hydrogen bond

The CP/LS NPs-incorporated hydrogels had good adhesiveness to different surfaces. As shown in Fig. 3a, the hydrogel strongly adhered to human skin and easily peeled off without leaving a residue. The strong adhesive strength allowed the hydrogel to be stretched to seven times of its original length from a steel surface without detachment. Moreover, the hydrogel could adhere to various surfaces such as glass, plastic, and animal tissue. Using the PEDOT/LS NPs as an example, the adhesion of PAM, PEDOT-PAM hydrogel, and PEDOT/LS-PAM hydrogel was compared to demonstrate the effect of the CP/LS NPs on the adhesiveness of the hydrogels. Figure 3b shows the adhesive strength of the PEDOT/LS-PAM hydrogel to steel (23.2 kPa), polytetrafluoroethylene (PTFE) (22.5 kPa), glass (21.5 kPa), and porcine skin (20 kPa). Hydrogels without CP/LS NPs did not exhibit effective adhesiveness. The content of PEDOT/LS NPs can affect the hydrogel adhesive strength (Fig. S5). The adhesive strength increased with increasing CP content because of the higher content of catechol groups in the hydrogel. The high adhesive strength of the PEDOT/LS-PAM hydrogel was maintained after 30 peeling-adhering cycles, which indicated that the CP/LS NPs-based hydrogels have repeatable adhesiveness (Fig. 3c). The adhesiveness of the NP-incorporated hydrogel was caused by the abundant catechol groups on the redox-active CP/LS NPs. XPS analysis proved that lignin had high contents of C-O and C–OH groups at 286.4 eV and a low content of C = O at 288.0 eV (Fig. S6). The high content of C–OH confirmed the presence of the catechol groups in the PEDOT/LS-PAM hydrogel [1, 46]. The catechol groups exert strong adhesion to various substrates through hydrogen bonds, coordination bond, covalent linking, and π–π interaction [32, 46] (Fig. 3d).

**Fig. 4:**
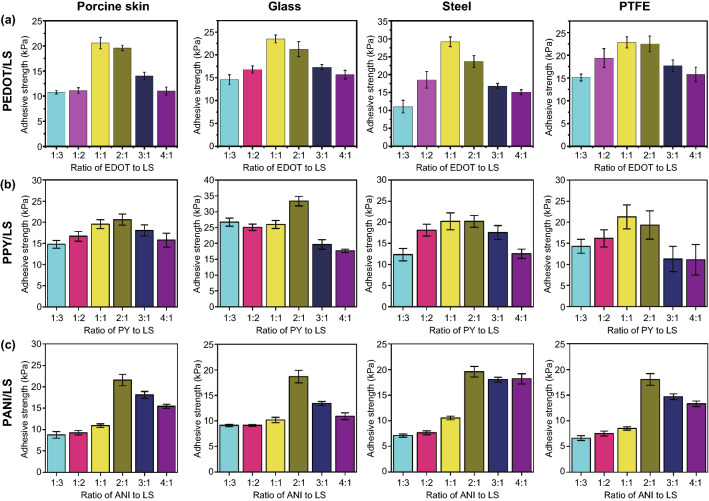
The effect of the mass ratio of CP to LS of the CP/LS NPs on the adhesive strength of the NPs-incorporated hydrogels **a** PEDOT/LS, **b** PPY/LS, and **c** PANI/LS. The content of the CP/LS NPs was 3 wt ‰

The adhesive strength of the hydrogels could be tuned by varying the ratio of CPs to LS. Figure 4 shows that the optimal mass–feed ratios in a range from 1:3 to 4:1 for the PEDOT/LS, PPY/LS, and PANI/LS hydrogels were 1:1, 2:1, and 2:1, respectively. This could be explained from two aspects. First, a low ratio of CP to LS may cause less electrons to maintain the redox balance of catechol/quinone groups. Second, a high ratio of CP to LS may result in insufficient catechol groups. Thus, only the NPs with a suitable ratio of CP to LS could possess enough catechol groups to endow the hydrogel with strong adhesion. In the following studies, we took PEDOT/LS-PAM hydrogels as representative samples.

### Bioelectronic Applications of the Hydrogels

**Fig. 5:**
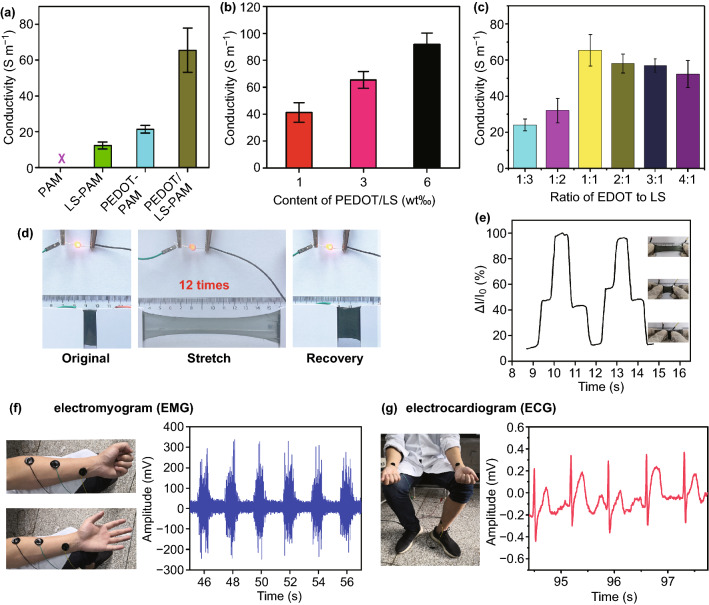
Conductivity and bioelectronic applications of the CP/LS NPs-incorporated hydrogels. **a** Conductivity of various hydrogels. (The content of various hydrogels is listed in Table S2.) **b** The content of the NPs affected the conductivity of the hydrogel. (The ratio of EDOT to LS of the NPs was 2:1.) **c** The ratio of EDOT to LS of the EDOT/LS NPs affected the conductivity of the hydrogel. (The content of NPs was 3 wt ‰.) **d** PEDOT/LS-PAM hydrogel was linked into a circuit to light a LED and stretched 12 times of its initial length. **e** PEDOT/LS-PAM hydrogel acted as a strain senor, and the tensile strain of hydrogel was monitored by measuring the electric current change during stretching and recovery. The PEDOT/LS-PAM hydrogels adhered to skin and acted as bioelectrodes to measure the biosignals of one of the authors, **f** electromyogram (EMG), **g** electrocardiogram (ECG)

The CP/LS NPs-incorporated hydrogel had good conductivity, which was attributed to the hydrophilicity of CP/LS NPs. With the hydrophilicity, the CP/LS NPs readily dispersed in the hydrogel network and formed well-connected electric pathways, endowing the hydrogel with good conductivity. The PEDOT/LS-PAM hydrogel had higher conductivity (68 S m^−1^) than that of the PEDOT-PAM hydrogel (21 S m^−1^) and LS-PAM hydrogel (12 S m^−1^) (Fig. 5a). Moreover, the conductivity of the hydrogel increased with increasing PEDOT/LS NP content (Fig. 5b). Furthermore, the ratio of CP to LS affected the conductivity of the CP/LS-PAM hydrogel (Figs. 5c and S7). When the ratio of CP (EDOT, PY, ANI) to LS was 1:1 or 2:1, the conductivity of the CP/LS-PAM hydrogel reached the maximum value.

Owing to the good conductivity and adhesiveness, the CP/LS NPs-incorporated hydrogel can be used for bioelectronic applications. The PEDOT/LS-PAM (EDOT/LS ratio = 2:1; the content of NPs is 3 wt‰) hydrogel was selected for following applications because PEDOT is electrochemically stable in aqueous solution [17]. The adhesive and conductive PEDOT/LS-PAM hydrogel adhered to plastic and was linked into a circuit with a light-emitting diode (LED) (Fig. 5d). The brightness of the LED varied with the strain of the hydrogel. The different strain state of the hydrogel was indicated by the increased resistance, and therefore, the hydrogel can act as a strain sensor (Fig. 5e). Meanwhile, the PEDOT/LS-incorporated hydrogels can be also used as the adhesive bioelectrodes to detect the electromyographic (EMG) and electrocardiographic (ECG) signals (Fig. 5f, g). In summary, this water dispersible CP/LS NPs-incorporated hydrogel has more stable conductivity, biocompatibility, and adhesiveness, which is a promising candidate for bioelectronics.

### Mechanical Properties of the Hydrogels

**Fig. 6:**
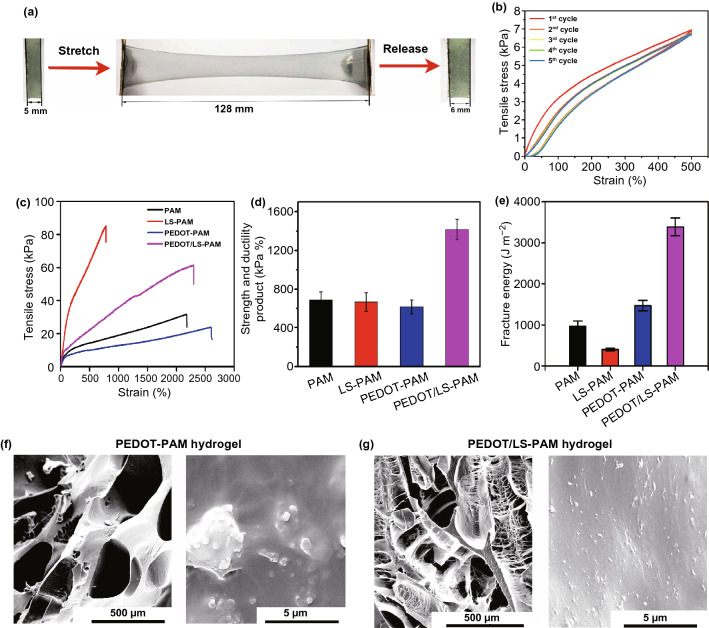
Mechanical properties and microstructures of hydrogels. **a** PEDOT/LS-PAM hydrogel with 3 wt ‰ of PEDOT/LS NPs was elongated to 25.6 times of its initial length and recovered. **b** Cyclic tensile loading–unloading curves of the PEDOT/LS-PAM hydrogel. Typical tensile stress–strain curves of **c** various hydrogels and **d** hydrogels with different contents of PEDOT/LS NPs. **e** Fracture energy of various hydrogels. Morphologies and magnified images of freezing dried hydrogels. **f** PEDOT-PAM hydrogel, and **g** PEDOT/LS-PAM hydrogel

The PEDOT/LS NPs endowed the hydrogel with good stretchability and recoverability. Figure 6a shows that the PEDOT/LS-PAM hydrogel nearly recovered to its original length after stretching. Cyclic loading–unloading tensile testing at a strain of 500% indicated that the first unloading path had a hysteresis loop with a small residual strain (Fig. 6b). This is similar to the typical behavior of elastomers [50]. In latter cycles of the loading–unloading tensile test, the PEDOT/LS-PAM hydrogel showed remarkable overlap in the cyclic tensile curves, indicating excellent elasticity and mechanical stability. Compared with the LS and PEDOT NPs, incorporating the PEDOT/LS NPs significantly improved the tensile strength (Figs. 6c and S8). The strength and ductility product (SDP) of various hydrogels indicated that the comprehensive mechanical performance of the PEDOT/LS-PAM hydrogel was improved by incorporation PEDOT/LS NPs in the hydrogel matrix (Fig. S8b). Increasing the content of PEDOT/LS NPs increased the tensile strength of the PEDOT/LS-PAM hydrogel (Fig. 6d). The PEDOT/LS-PAM hydrogel could be stretched to 25 times its initial length with a strength of 61.5 kPa, when the NPs content was 3 wt%. However, the high content of NPs (6 wt%) decreased the stretchability of the hydrogel, which was caused by aggregation of NPs in the hydrogel network. The fracture energy of the PEDOT/LS-PAM hydrogel (3500 J m^−2^) was higher than that of the PAM hydrogel (1000 J m^−2^), LS-PAM hydrogel (500 J m^−2^), and PEDOT-PAM hydrogel (1500 J m^−2^) (Fig. 6e). The high toughness of the hydrogel was attributed to the nanoreinforcement effects of the incorporated NPs. These NPs were uniformly distributed in the hydrogel and introduced noncovalent interactions into the chemically cross-linked PAM hydrogel and consequently increased the energy dissipation ability [51, 52]. As revealed by SEM, the lyophilized bare PAM hydrogel showed large pores (Fig. S9). After incorporating PEDOT, the hydrogel exhibited a porous structure and PEDOT aggregated in the hydrogel network (Fig. 6f). In contrast, the lyophilized PEDOT/LS-PAM hydrogel exhibited an interwoven microfibril structure and the PEDOT/LS NPs were uniformly distributed within the hydrogel (Fig. 6g).

**Fig. 7:**
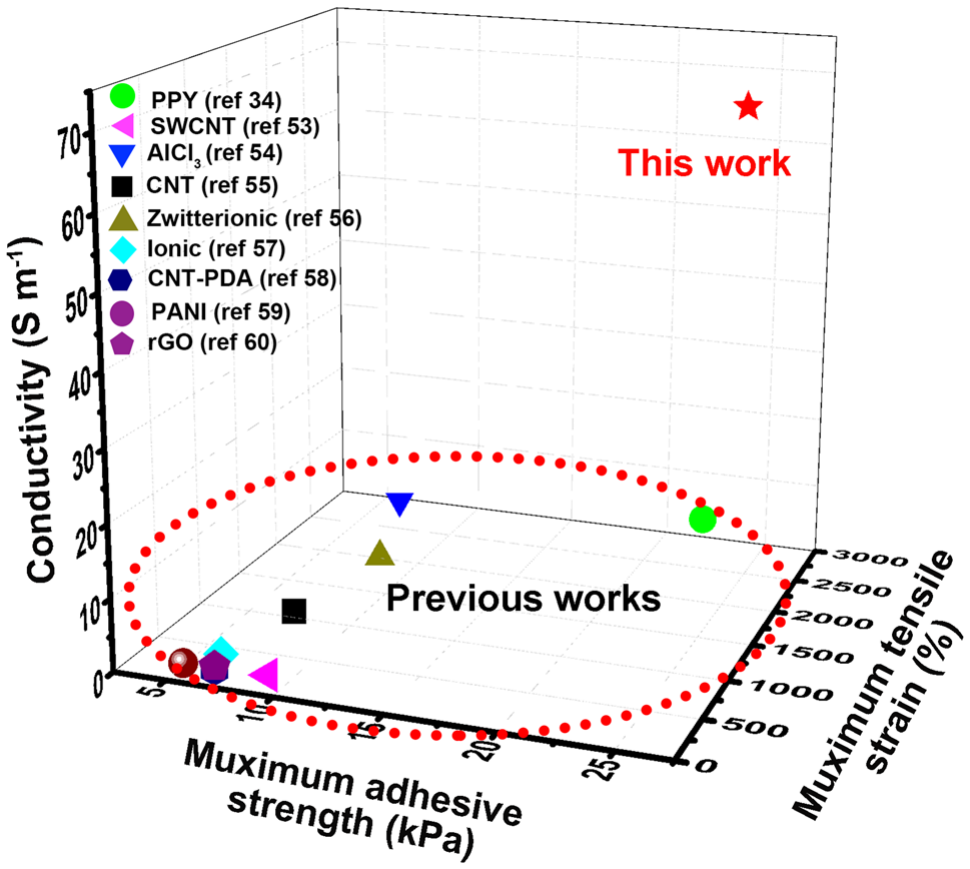
Comparison of this work with previous reported conductive hydrogels in terms of stretchability, adhesiveness, and conductivity

Compared with the previously reported typical conductive hydrogels (Fig. 7), the PEDOT/LS NPs-incorporated hydrogel has better adhesiveness, mechanical properties, and conductivity, which is an ideal material for bioelectronic applications [34, 53–60]. First, the hydrogel exhibits long-term and repeatable adhesiveness because the redox activity of PEDOT/LS NPs endows the hydrogel with catechol groups, which avoids interfacial delamination and reduces interfacial resistance between the hydrogel and the contacted human skin during biosignal detection. In particular, the hydrogel possesses suitable adhesive properties to skin surfaces and can be easily peeled off without any residue and anaphylactic reaction. Second, the hydrogel has good conductivity because the hydrophilic PEDOT/LS NPs form well-connected conductive pathways in the hydrogel network. Consequently, the hydrogel exhibits excellent sensor performance for ultrasensitive healthcare monitoring. Third, the hydrogel has good mechanical properties because the PEDOT/LS NPs introduce noncovalent bonds and nanoreinforcement effect into the chemical cross-linked hydrogel networks. Thus, the hydrogel could suffer mechanical deformation during the biosignal detection.

### Electrostimulated Cell Culture

**Fig. 8:**
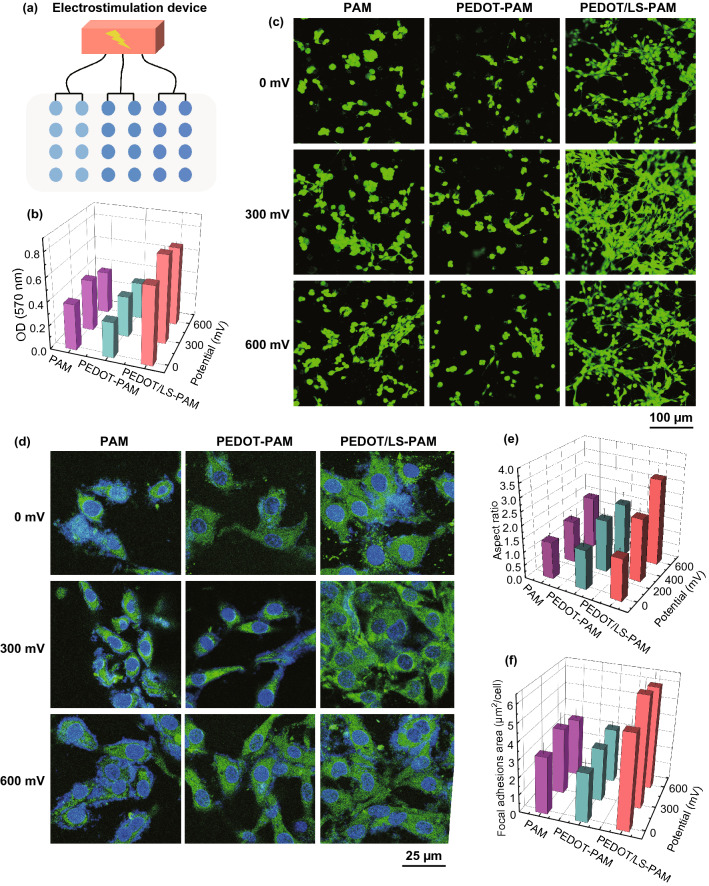
Electrostimulation of C2C12 cells on different hydrogels after 7 days of culture. **a** Homemade multi-channel high-throughput cell electrical stimulation device. **b** Proliferation of C2C12 measured by MTT analysis. **c** Fluorescent micrographs of C2C12 cultured on different hydrogels under electrostimulation. Cells were stained by Calcein-AM (Green). **d** Focal adhesion formation of C2C12 cells on different hydrogels under electrostimulation. Cell stained with DAPI for nuclei (blue) and monoclonal antibody for focal adhesions (green). **e** Aspect ratio of C2C12. **f** Focal adhesion size per cell

The hydrogel can be used to regulate cell behavior through electrical stimulation due to its good conductivity and cell adhesiveness. The proliferation and adhesion spreading of C2C12 were evaluated on the PAM, PEDOT-PAM, and PEDOT/LS-PAM hydrogels with a homemade high-throughput electrostimulation device (Fig. 8a) under electrostimulation voltages of 0, 300, and 600 mV. Compared with PAM and PEDOT-PAM hydrogels, cells on the PEDOT/LS-PAM hydrogel exhibited better proliferation activities. However, the proliferation activity of C2C12 decreased under the higher potential (Fig. 8b). Cell spreading and focal adhesion formation on the different hydrogels indicated that the spreading and adhesion of C2C12 on the PEDOT/LS-PAM hydrogel were better than that on the PEDOT-PAM and PAM hydrogels (Fig. 8c, d). Vinculin is a highly conserved actin-binding protein that is frequently used as a marker for focal adhesion [61, 62]. Vinculin was stained to reveal the cell adhesion (Fig. 8d). Cells grown on PAM and the PEDOT-PAM hydrogel showed few linking filaments and reduced spreading. Cells on the PEDOT/LS-PAM hydrogel were more clustered with extensive actin filaments linking adjacent cells. Electrostimulation increased the size of the focal adhesions when the potential was lower than 300 mV. The aspect ratio of C2C12 indicated the earliest stage of myotubes formation [63, 64]. The C2C12 on the PEDOT/LS-PAM hydrogels were more elongated than those on the PAM and PEDOT-PAM hydrogel under electrostimulation voltages of 600 mV (Fig. 8e). Electrostimulation increased the size of the focal adhesions when the potential was lower than 300 mV (Fig. 8f). In short, the results of electrostimulated cell culture indicated that the PEDOT/LS-PAM hydrogel has good biocompatibility and conductivity and therefore can act as a bridge for promoting the transduction of the physiological electrical signals. Previous studies proved that the CH has ability not only to regulate cell adhesion and proliferation, but also to improve the expression of the factors related to the tissue regeneration [5, 64]. Thus, the conductive PEDOT/LS-PAM hydrogel has potential in cell stimulation and related bioelectronic applications at cellular level.

### In vivo Biocompatibility of the Hydrogels

**Fig. 9:**
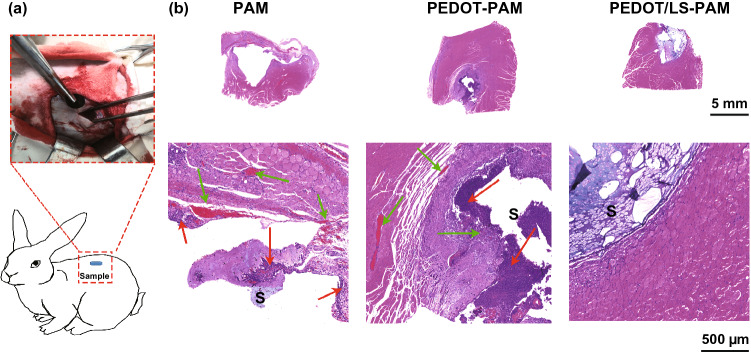
a Surgical operation of the hydrogel implanted in subcutaneous muscle space of New Zealand white rabbit for biocompatibility evaluation. **b** Representative photomicrographs of hematoxylin and eosin (H&E) stained surrounding tissues after 14 days of implantation. Green arrows indicate eosinophils; red arrows indicate macrophages; S means sample

In vivo biocompatibility is of critical importance for bioimplantable applications of the hydrogels. To evaluate the in vivo biocompatibility, the PAM, PEDOT-PAM, and PEDOT/LS-PAM hydrogels were implanted into subcutaneous muscle spaces of New Zealand white rabbits and retrieved after 14 days (Fig. 9a). Histological staining revealed that the PAM hydrogel was surrounded by a thin inflammatory zone with eosinophils (green arrow) and macrophages (red arrow) (Fig. 9b). For the PEDOT-PAM hydrogel, a thick reactive inflammatory area was observed. The inflammatory area was surrounded by many macrophages, neutrophils, and eosinophils. In contrast, the PEDOT/LS-PAM hydrogel exhibited minimal inflammatory reaction and integrated with the surrounding muscle tissue because of the existence of the tissue affinitive catechol groups. Thus, the PEDOT/LS-PAM hydrogel is a potential material for bioimplantable applications.

## Conclusion

In conclusion, we developed a universal strategy to prepare hydrophilic, redox-active, and biocompatible CP/LS NPs. The CP/LS NPs were used as nanofillers for construction of conductive and adhesive hydrogels. The CP/LS NPs significantly improved the conductivity of the hydrogel due to their water dispersibility and consequent uniform distribution in the hydrogel networks. The CP/LS-incorporated hydrogel had long-term and repeatable adhesive properties, which were attributed to the dynamic redox balance of catechol/quinone groups of the CP/LS NPs. Meanwhile, the mechanical properties of the hydrogel were also enhanced by the CP/LS NPs, which was attributed to nanoreinforcement effects and noncovalent interactions between the NPs and chemically cross-linked PAM network.

With the good conductivity, adhesiveness, and mechanical properties, the hydrogel was used as a flexible and adhesive strain sensor and bioelectrode for monitoring biosignal. Moreover, the PEDOT/LS-PAM hydrogel showed good biocompatibility and electroactive properties favoring cell spreading/growth and therefore has potential in the electrostimulation of tissue regeneration as the implantable bioelectrodes. This is because the implantation of electroactive materials promoted the transmission of physiological electrical signals among the cells and enhanced the activity of cells [65, 66]. This universal strategy for preparing redox-active, hydrophilic, and conductive NPs is a breakthrough to overcome the intrinsic shortcomings of CPs, such as hydrophobicity and brittleness. Compared with previous simple blending, this strategy of in situ forming nanostructures initiates a new route to employ CPs into hydrogels for flexible and adhesive bioelectronic devices.

## Electronic Supplementary Material

Below is the link to the electronic supplementary material.

Supplementary material 1 (PDF 717 kb)
