# Supplementary material for: Mussel-Inspired Redox-Active and Hydrophilic Conductive Polymer Nanoparticles for Adhesive Hydrogel Bioelectronics
Source: Nanomicro Lett. 2020 Aug 18;12:169. doi: 10.1007/s40820-020-00507-0 (PMC7770971; doi:10.1007/s40820-020-00507-0)
Supplement: Supplementary file 1 — Supplementary material 1 (PDF 717 kb) [file 40820_2020_507_MOESM1_ESM.pdf]

Supporting Information for

Mussel-Inspired Redox-Active and Hydrophilic Conductive Polymer Nanoparticles for Adhesive Hydrogel Bioelectronics

Donglin Gan[1, †], Tao Shuai[1, †], Xiao Wang[1], Ziqiang Huang[1], Fuzeng Ren[2], Liming Fang[3], Kefeng Wang[4], Chaoming Xie[1, *], Xiong Lu[1,]

[1]Key Lab of Advanced Technologies of Materials, Ministry of Education, School of Materials Science and Engineering, Southwest Jiaotong University, Chengdu 610031, People's Republic of China

[2]Department of Materials Science and Engineering, Southern University of Science and Technology, Shenzhen, Guangdong 518055, People's Republic of China

[3]Department of Polymer Science and Engineering, School of Materials Science and Engineering, South China University of Technology, Guangzhou, People's Republic of China

[4]National Engineering Research Center for Biomaterials, Sichuan University, Chengdu 610064, People's Republic of China

†Donglin Gan and Tao Shuai contributed equally to this work.

*Corresponding authors. E-mail: xie@swjtu.edu.cn (C. Xie); luxiong_2004@163.com (X. Lu)

S1 Preparation of Sulfonated Lignin

Lignin was sulfonated by APS according to the previous report [S1, S2]. First, an aqueous solution of lignin at a concentration of 0.1 g mL^{-1} was prepared by dissolving the weighed amount of lignin powder in an NaOH solution (pH = 11) with the aid of ultrasonic agitation. Then, APS solution (0.6 g mL^{-1}) was added dropwise into the above mixture in an oil bath for 3 h. The reaction mixture was then cooled to room temperature. The LS solution was obtained by filtering with a 0.5 μm filter.

Table S1 Content of various CP/LS NPs, three kinds of conductive polymer (CP), such as PEDOT, PPY and PANI were used for preparation of CP/LS NPs

CP:LS	CP (g)	LS (g)	APS (g)	DI Water (mL)
CP	0.3	0	0.45	10
CP:LS=1:3	0.1	0.3	0.15	10
CP:LS=1:3	0.15	0.3	0.225	10
CP:LS=1:1	0.3	0.3	0.45	10
CP:LS=2:1	0.6	0.3	0.9	10
CP:LS=3:1	0.9	0.3	1.35	10
CP:LS=4:1	1.2	0.3	1.8	10

S2 Characterization of CP/LS NPs

S2.1 Morphologies of the CP/LS NPs

The morphologies of the CP/LS NPs were examined by scanning electron microscopy (SEM,

JSM 6390, JEOL, Japan).

S2.2 X-ray Photoelectron Spectroscopy (XPS) Analysis of CP/LS NPs

XPS analysis was used to monitor the variations in the catechol groups of PSGO and the PSGO-PEDOT nanosheets. The nanosheets were freeze-dried prior to the analysis. A monochromatic

c Al Kα X-ray excitation source (hν = 1486.6 eV) was used at 15 KV and 150 W, and the C1s spectra at the binding energy of 285 eV was set as the reference.

Table S2 Content of various hydrogel

Hydrogel	CP/AM (wt%)	CP/LS/AM (wt%)	AM (g)	APS/AM (wt%)	BIS/AM (wt%)	TMEDA (μL)	DI Water (mL)
PAM[1]	0	0	2.6	2	0.4	25	10
CP-PAM[1,*]	0.3	0	2.6	2	0.4	25	10
1‰CP/LS-PAM[*]	0	0.1	2.6	2	0.4	25	10
3‰CP/LS-PAM[1,*]	0	0.3	2.6	2	0.4	25	10
6‰CP/LS-PAM[*]	0	0.6	2.6	2	0.4	25	10

1. PAM, PEDOT-PAM and PEDOT/LS-PAM hydrogels used for cell culture and animal experiments.

* PEDOT, PPY and PANI were used for preparation of CP/LS NPs.

S2.3 Antioxidative activity of the PEDOT/LS NPs

The antioxidative activity of the PEDOT/LS NPs were tested by measuring their capacities to scavenge stable DPPH free radicals using an established method with minor modifications[3]. First, a homogenate of the PEDOT/LS NPs (1 mg/mL) was prepared by grinding and dispersal in methanol. Next, 1,1-diphenyl-2-picrylhydrazyl (DPPH, 3.0 mL, 100 μM) was added, and then the mixture was incubated in dark for 30 min. Afterwards, wavelength scanning of the mixture was performed using a UV-vis spectrophotometer (TU-1901, Puxi, China). The capacity of the PEDOT/LS NPs to scavenge DPPH free radicals was evaluated using the following equation:

$$\text{DPPH scavenging}\,\% = \frac{A_B - A_H}{A_B} \times 100 \qquad \text{(S1)}$$

where A_B is the absorption of the blank (DPPH + methanol) and A_H is the absorption of the PEDOT/LS NPs (DPPH + methanol + PEDOT/LS NPs).

S3 Characterization of the Hydrogels

S3.1 Morphologies of the Freeze-dried Hydrogels

The fresh hydrogel samples were freeze-dried at −80 ºC. The freeze-dried PEDOT-PAM and PEDOT/LS-PAM hydrogels were then broken apart and their inner morphologies were observed by SEM (JSM 6300, JEOL, Japan).

S3.2 Adhesive Property of the Hydrogels

The adhesive strength of the PAM, PEDOT-PAM, and PEDOT/LS-PAM hydrogels were measured by carrying out their tensile adhesive tests according to a previously reported procedure [S4]. The hydrogels with a bonded area of 25×25 mm^2 were applied to the surface

of the specimens. The samples were pulled to failure at a crosshead speed of 5 mm min^{-1} using a universal testing machine (Instron 5567, USA) equipped with a 100 N-load cell under ambient conditions until their separation.

S3.3 Conductivity of the Hydrogels

Two-probe method on an electrochemical system was used to measure the conductivity of the hydrogels (CHI 660, Chenghua, China) according to a previously reported methods[5]. Briefly, the hydrogels (R = 7.5 mm, L = 10 mm) were placed between two parallel titanium electrodes, which were connected by an electrical loop. Different electrical currents (I) (0.001, 0.002, 0.003, 0.004, and 0.005 A) were applied to the hydrogel and the electrical potentials (V) were recorded. The conductivity σ of the hydrogels was calculated using Eq. S2:

$$\sigma = \frac{IL}{V*\pi R^2} \qquad \text{(S2)}$$

where L and R are the length and diameter of the hydrogels, respectively.

S4 Bioelectronics Applications of PEDOT/LS-PAM Hydrogels

The PEDOT/LS-PAM hydrogels (D = 20 mm, H = 2 mm) were used as the adhesive hydrogel strain sensors and electrodes for electromyogram (EMG), electrocardiogram (ECG) recording. For the EMG recording, the hydrogel was adhered on the arm of the author. For the ECG recoding, the hydrogel was adhered on the arms and the left medial leg. The EMG and ECG data were recorded by using a multichannel physiological signal acquisition and process system (RM620C, Chengdu Instrument factory, China).

To demonstrate the sensing capability of the PEDOT/LS-PAM hydrogels, they were adhered to human wrist to monitor the tensile behaviour of the hydrogels. The variations of current caused by the deformation of the hydrogels were recorded using an electrochemical system with the amperometric i-t curve technique (CHI 660C, Chenhua, China).

S5 Mechanical Properties of the Hydrogels

The mechanical properties of the hydrogels were evaluated using a universal test machine (Instron 5567, USA) according to the previously reported procedures [S6]. The tensile tests of the specimens (width: 25 mm, length: 5 mm, thickness: 2 mm) were carried out at an extension speed of 10 mm min^{-1}.

The strength and ductility product (SDP) is an indicator of the comprehensive mechanical performance to characterize the levels of strength and toughness at a static state. To demonstrate the good tensile properties of PEDOT/LS hydrogels, the SDP was calculated according the following equation:

SDP (MPa %) = Maximum tensile strength (MPa) × Maximum tensile strain (%)   (S3)

S6 Fracture Energy Test

The fracture energy was determined by the standard single-edge notch test, using a universal testing machine (Instron 5567) according to previous report [S5, S7]. The crosshead speed was 2 mm s^{-1} and the specimens were fixed between clamps with a gauge length of 5 mm. The thickness and width of the specimens were 2 and 20 mm, respectively. Two specimens

(one notched and the other unnotched) were pulled during the test. The unnotched specimen was pulled to obtain the stress-strain curve, whereas the notched one was used to determine the critical extension ratio (λc), at which cracks expanded. A notch of 5-mm length was introduced in the middle of the notched specimen. The fracture energy (J m^{-2}) was calculated using the following equation, proposed by Greensmith for elastomers [S7]:

$$Gc = \frac{1}{B}\left[\frac{\partial(\Delta U)}{\partial a}\right] = 2\frac{\pi}{\sqrt{\lambda c}}aw_0 \qquad (S4)$$

where a is the length of the crack and λc is the extension ratio at which cracks expand in the single-edge notch tests.

S7 In vitro Evaluation of the Cytocompatibility of the Hydrogels

The cytocompatibility of the PAM, PEDOT-PAM and PEDOT/LS-PAM hydrogels (D = 8 mm, H = 2 mm) was evaluated using C2C12 cells (Stem Cell Bank, Chinese Academy of sciences) according to the previous study [S4, S6]. Before cell culture, the samples were purified by repeatedly deswelling in pure ethanol and swelling in PBS, at least three cycles. They were further sterilized by 75% ethanol for a week. The sterilized samples were then placed into a 48-well plate. Cells (5×10^4 cell/sample) were seeded onto the hydrogels and were left undisturbed in a CO_2 incubator at 37 °C for 2 h to allow cell attachment. Then, 1 mL of DMEM supplemented (HyClone, USA) with 10% fetal bovine serum (BI, USA) and 1 % penicillin-streptomycin solution (HyClone, USA) was added into each well. The cells were allowed to adhere and culture for 7 days. Cell proliferation was evaluated by MTT assay. Calcein AM staining was used to observe the cell viability on the hydrogels. In addition, the focal adhesion formation was assessed by rabbit monoclonal antibody to vinculin (ab196454, Abcam, UK) and 4,6-diamidino-2-phenylindole (DAPI, Abcam, UK) after three days' culture. The cells were examined using a confocal laser scanning microscope (CLSM, Leica, Germany). The aspect ratio and focal adhesion area was determined using Image J software. When two focal adhesions overlapped or were in close proximity to each other, they were considered as distinct if the intensity profile revealed that the intensity at the trough between the two focal adhesions was less than 60 % of the average intensity of the two peaks.

A multi-channel cell electrostimulation (ES) device developed in-house was used to carry out the ES experiments of the cells[6]. The ES experiments were carried out when the cells were seeded on the hydrogels after 1 day. Various ES voltages (0, 300, and 600 mV) were applied on the cells for 30 min every day. After 7 days of stimulation, the proliferation was assayed to examine the electrical response of C2C12 on the hydrogels.

S8 In vivo Evaluation of the Biocompatibility of the Hydrogels

All the animal experiments were performed according to the protocols approved by the local ethical committee and the laboratory animal administration rules of China. The surgical procedures were carried out as reported previously [S4]. For biocompatibility studies, four New Zealand white rabbits of either gender (about 3 kg) were sedated with pentobarbital (3 wt%, 40 mg kg^{-1}) and a section of fur on their back was shaved. The four parallel sterilized PAM, PEDOT-PAM and PEDOT/LS-PAM hydrogels (D = 5 mm, H = 3 mm, total 12 samples) were separately implanted into different subcutaneous muscle spaces. On the 14th day after the surgery, the subcutaneous muscle tissues surrounding the samples were harvested. The harvested muscle tissues were fixed in a 10% neutral buffered formalin solution for 24 h and were stained with hematoxylin and eosin (H&E) for evaluating the

biocompatibility of the hydrogels.

S9 Supplementary Figures

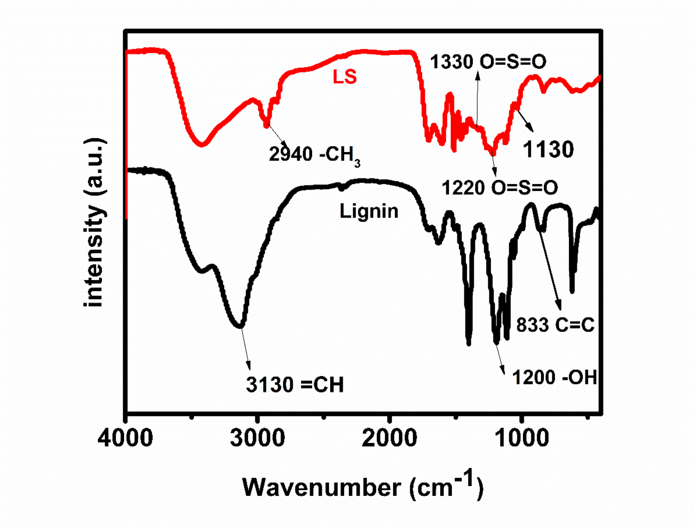

Fig. S1 FTIR spectra of lignin and lignosulfonates (LS)

The FTIR analysis was conducted to prove that the lignin was sulfonated by APS successfully. The FTIR spectra of lignin presented typical band information, such as aromatic (1630 cm^{-1}), aliphatic (1510 cm^{-1}), C=C (833 cm^{-1}, 3130 cm^{-1}) and -OH (1200 cm^{-1}, 3420 cm^{-1}). The peak of S=O (1040 cm^{-1}) only appeared in the LS and was assigned to $-SO_3$ groups, indicating the successful decoration of LS.

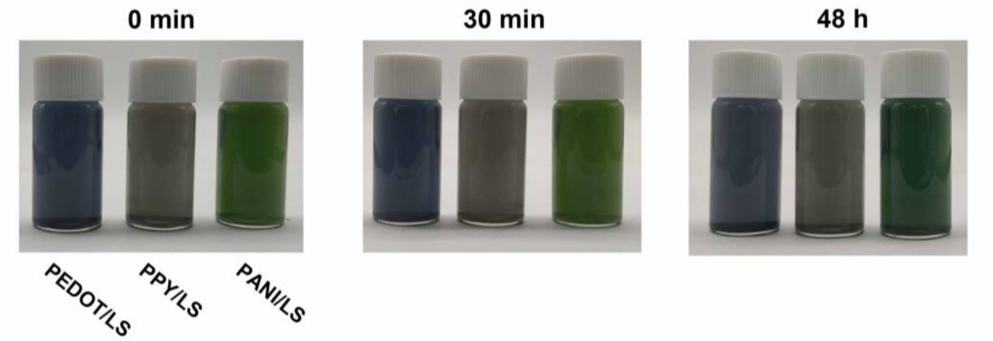

Fig. S2 Dispersibility of the PEDOT/LS, PPY/LS and PANI/LS NPs in water

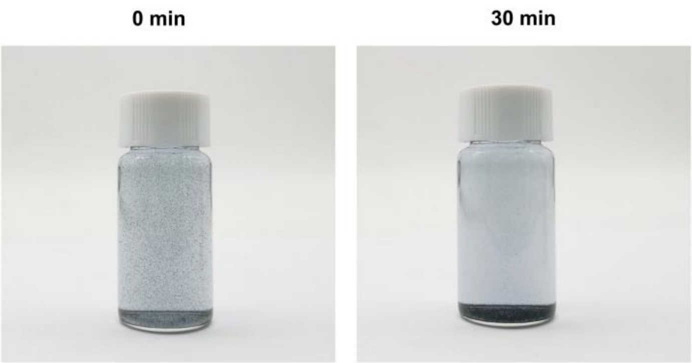

Fig. S3 Dispersion of the pristine PEDOT NPs in water

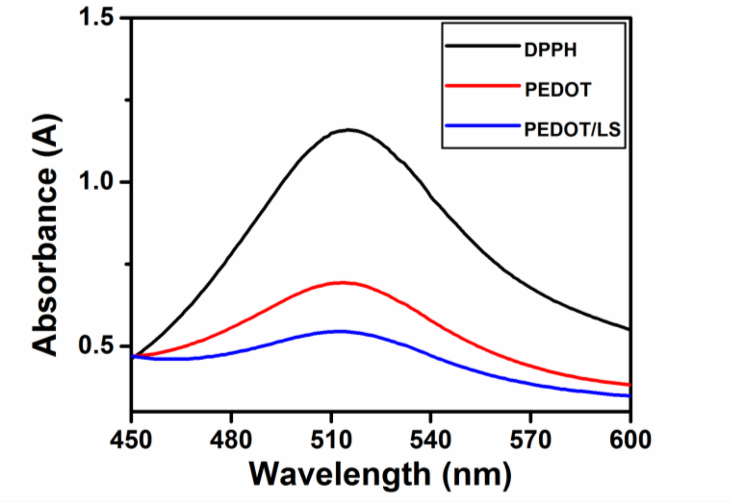

Fig. S4 UV-vis spectra of DPPH and scavenging by PEDOT NPs and PEDOT/LS NPs

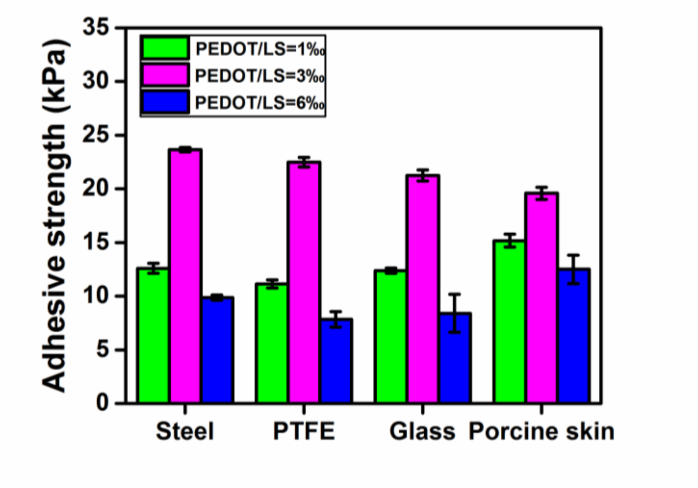

Fig. S5 Adhesive strength of the PEDOT/LS hydrogel with different contents of PEDOT / LS NPs. The mass ratio of PEDOT to LS is 2:1

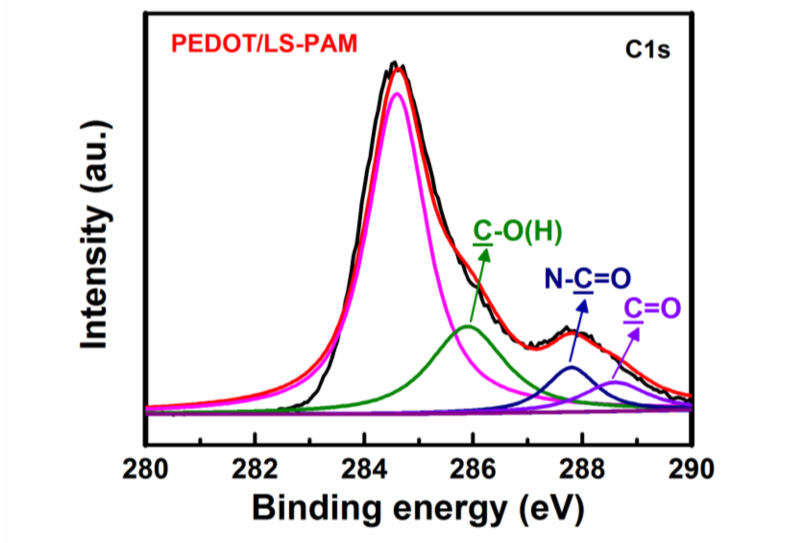

Fig. S6 High-resolution XPS spectra of C1s and their deconvolution

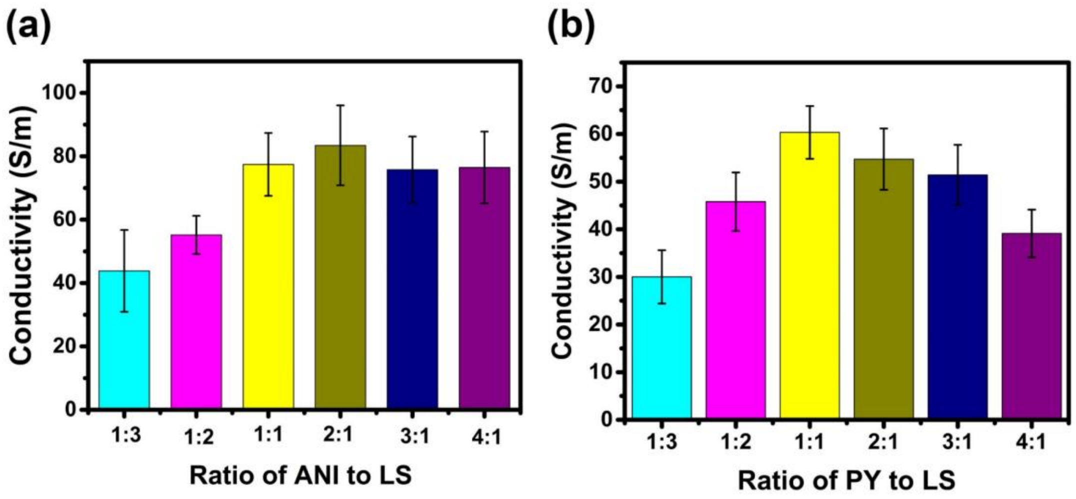

Fig. S7 Conductivity of hydrogels with different ratio of CP to LS, (**a**) ANI to LS, (**b**) PY to LS. The content of the NP is 3 wt‰

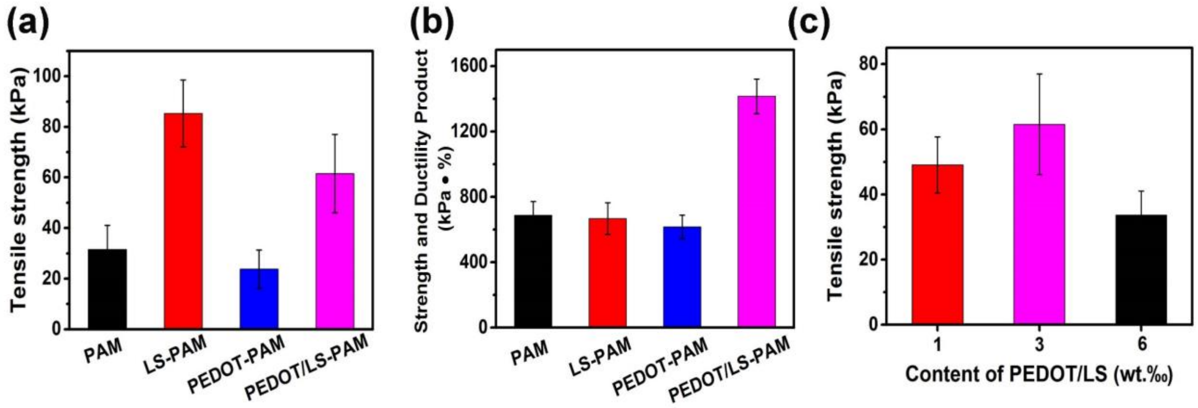

Fig. S8 (**a**) Tensile strength of various hydrogels. (**b**) Strength and ductility product of various hydrogels. (**c**) Tensile strength of hydrogels with different content of PEDOT/LS (EDOT:LS= 2:1)

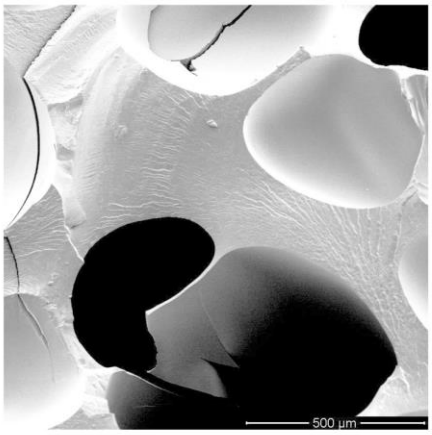

Fig. S9 SEM image of pure PAM hydrogel after freezing dry

Supplementary References

[S1]F. Li, X. Wang, R. Sun, A metal-free and flexible supercapacitor based on redox-active lignosulfonate functionalized graphene hydrogels. J. Mater. Chem. A **5**(39), 20643-20650 (2017). https://doi.org/https://doi.org/10.1039/C7TA03789A

[S2]F. Ajjan, N. Casado, T. R bi , A. Elfwing, N. Solin et al., High performance pedot/lignin biopolymer composites for electrochemical supercapacitors. J. Mater. Chem. A **4**(5), 1838-1847 (2016). https://doi.org/https://doi.org/10.1039/C5TA10096H

[S3]X. Shen, P. Berton, J. L. Shamshina, R. D. Rogers, Preparation and comparison of bulk and membrane hydrogels based on kraft-and ionic-liquid-isolated lignins. Green Chem. **18**(20), 5607-5620 (2016). https://doi.org/https://doi.org/10.1039/C6GC01339B

[S4]D. Gan, Z. Huang, X. Wang, L. Jiang, C. Wang et al., Graphene oxide-templated conductive and redox-active nanosheets incorporated hydrogels for adhesive bioelectronics. Adv. Funct. Mater. 1907678 (2019). https://doi.org/https://doi.org/10.1002/adfm.201907678

[S5]D. Gan, L. Han, M. Wang, W. Xing, T. Xu et al., Conductive and tough hydrogels based on biopolymer molecular templates for controlling in situ formation of polypyrrole nanorods. ACS Applied Mater. Interfaces **10**(42), 36218-36228 (2018). https://doi.org/https://doi.org/10.1021/acsami.8b10280

[S6]D. Gan, L. Han, M. Wang, W. Xing, T. Xu et al., Conductive and tough hydrogels based on biopolymer molecular templates for controlling in situ formation of polypyrrole nanorods. ACS Appl. Mater. Interfaces **10**(42), 36218-36228 (2018). https://doi.org/https://doi.org/10.1021/acsami.8b10280

[S7]L. Han, X. Lu, M. Wang, D. Gan, W. Deng et al., A mussel-inspired conductive, self-adhesive, and self-healable tough hydrogel as cell stimulators and implantable bioelectronics. Small **13**(2), 1601916 (2017). https://doi.org/https://doi.org/10.1002/smll.201601916
